# Using Clustered Regularly Interspaced Short Palindromic Repeats gene editing to induce permanent expression of fetal hemoglobin in β-thalassemia and sickle cell disease: A comparative meta-analysis

**DOI:** 10.3389/fmed.2022.943631

**Published:** 2022-09-29

**Authors:** Anthony Quagliano, Daniel Acevedo, Patrik Hardigan, Samiksha Prasad

**Affiliations:** Dr. Kiran C. Patel College of Allopathic Medicine, Nova Southeastern University, Fort Lauderdale, FL, United States

**Keywords:** β-thalassemia, BCL11A, HBG1/2, hemoglobinopathies, fetal hemoglobin (HbF), gene editing (CRISPR/Cas9), sickle cell disease

## Abstract

β-hemoglobinopathies like sickle cell disease (SCD) and β-thalassemia are characterized by differing mutations in the hemoglobin subunit beta gene (HBB). These disorders vary in phenotypic presentation and severity, with more severe manifestations leading to transfusion dependence along with associated complications such as infection and iron overload. β-hemoglobinopathies symptoms rapidly worsen after birth as the levels of fetal hemoglobin (HbF) begin to decline. To reverse this decline, current treatment plans typically involve the use of pharmacological agents such as hydroxyurea to raise expression levels of HbF. However, these treatments only result in transient effects and must be consistently administered. Gene editing technologies such as CRISPR/Cas9 (Clustered Regularly Interspaced Short Palindromic Repeats- CRISPR associated protein) offer the opportunity to create novel treatments which can raise HbF expression with potential permanent effects. Two gene targets, B-cell lymphoma/leukemia 11A gene (BCL11A) and the promoter regions of gamma globin genes (HBG1/2), have been identified to significantly increase HbF protein expression. In order to differentiate the effectiveness of BCL11A and HBG1/2 editing, a meta-analysis was performed by first identifying 119 studies for inclusion based on the search terms terms “β-Thalassemia,” “beta-thal” “sickle cell disease,” “SCD,” and “CRISPR.” Following application of exclusion and inclusion criteria, we performed analysis on 8 peer-reviewed published studies from 2018 to 2021 were included in the study. Forest plots were generated using R (version 4.1.2). Primary comparative analysis shows HBG1/2 had a significantly (*p* < 0.01)greater impact on induction of HbF expression compared to BCL11A. This analysis leads us to conclude that HBG1/2 merits further investigation as a possible gene editing target for treatment of SCD and β-thalassemia.

## Introduction/Background

The CRISPR (Clustered Regularly Interspaced Short Palindromic Repeats) – Cas (CRISPR-associated protein) systems are part of the adaptive immune system used by archaea and bacteria against foreign genetic elements (e.g., viruses or plasmids). The foreign genetic material is stored in the CRISPR sequence of the host’s genome and is referred to as a “spacer.” Different spacers are separated by short palindromic repeats. Spacers can be transcribed to form crRNA (CRISPR-RNA). crRNA is then attached to the Cas protein by trcrRNA (trans-activating CRISPR RNA), this complex is used to guide the Cas proteins to the foreign genetic material, which the nucleases (Cas) will then cleave (1–3).

This system can be manipulated by using different gRNAs (guide RNA; dual tracrRNA:crRNA chimera) (4) and different Cas proteins to potentially silence genes, fix mutated genes, or even embed new genes. β-hemoglobinopathies are among the most prevalently inherited disorders. With symptoms typically worsening after birth as fetal hemoglobin (HbF) levels decline, genome editing allows an interesting opportunity to alleviate these symptoms.

Disorders such as Sickle cell disease (SCD) or β-thalassemia are characterized by differing mutations in the hemoglobin subunit beta gene (HBB) on chromosome 11 (5, 6). While these β-hemoglobinopathies have a wide array of phenotypic manifestations, individuals who have more severe manifestations tend to have higher rates of morbidity and mortality (7, 8).

Sickle cell disease is caused by a single base substitution at the 6th codon of the HBB gene. A thymine is switched to an adenine, resulting in a glutamate (hydrophilic) being replaced with a valine (hydrophobic) (9, 10). This mutation, under hypoxic conditions, causes the red blood cells to have their characteristic rigid sickle shape, allowing them to stick to vessel walls and clump together (sickling). This sickling can lead to vaso-occlusive crises, also known as pain crises (6). SCD refers to the full range of potential genotypes involving the mutated HBB gene, not to be confused with sickle cell anemia which refers specifically to being homozygous for this mutated HBB gene.

While SCD is caused by a single point mutation, β-thalassemia has over 300 different reported mutations. Most of these mutations are single point mutations in different functionally important regions of the HBB gene. The frequency of these different mutations varies by population (11, 12). The severity of the illness caused by these respective mutations also varies (13). β-thalassemia is broken up into two types, minor and major. Carriers of a single mutated allele are considered to have β-thalassemia minor. People who are homozygous for the mutated alleles are said to have β-thalassemia major. The end result of these differing mutations is a decreased production of the β-globin chain (5).

**FIGURE 1:**
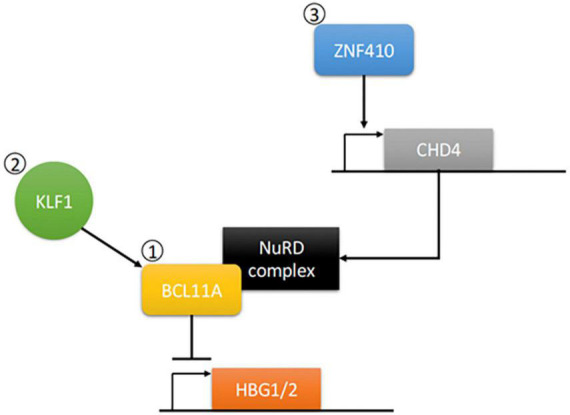
Control of Gamma Globin Gene Transcription. Gamma globin production for the generation of fetal hemoglobin (HbF) occurs through the transcription of the HBG1/2 genes. (1) This can be negatively regulated through expression of B-cell lymphoma/leukemia 11A gene (BCL11A), which has been extensively studied in CRISPR knockout models for the induction of gamma globin expression (Frangoul et al. (13), Lamsfus-Calle et al. (25), Khosravi et al. (18), Wu et al. (17). (2) Kruppel-like factor 1 (KLF1) can directly activate gamma globin gene repressors such as BCL11A to play a role in switch from gamma to beta globin. CRISPR knockout studies of KLF1 have showed induction of gamma globin expression [Shariati et al. (21)]. (3) Zinc finger protein 410 (ZNF410) can regulate BCL11A repression of gamma globin gene expression *via* induction of the Nucleosome Remodeling Deacetylase (NuRD) complex component CHD4. BCL11A contains a motif that can interact with a variety of NuRD-associated molecules, thus allowing for activation and then subsequent repression of gamma globin gene expression. CRISPR knockout studies of ZNF410 have identified its role as a possible gene target for induction of gamma globin expression [Lan et al. (20)]. Speckle-type POZ protein (SPOP) not included in the pathway.

Treatment for these disorders typically involves frequent blood transfusions. However, transfusion dependency has many associated complications such as infection or iron overload (12). Other treatments involve the use of pharmacological agents to raise the levels of fetal hemoglobin (HbF), which increases total hemoglobin production while also avoiding the use of the mutated β-globin chain in SCD (12). The effects of these treatments, however, are only transient. However, a number of genes have been identified that can control HbF expression (Figure 1). Thus, gene editing technologies allow the opportunity to create a more lasting treatment. In this paper, we will look at the use of CRISPR-Cas9 in inducing long-term expression of HbF, which has been shown to lower morbidity and mortality in β-hemoglobinopathies (14, 15).

The B-cell lymphoma/leukemia 11A gene (BCL11A) and promoters of the gamma globin genes (HBG1 and HBG2) have been largely studied for inducing long-term expression of HbF (16, 17). CRISPR studies involving these genes have had promising results thus far (18, 19). However, Krueppel-like factor 1 (KLF1), zinc-finger protein 410 (ZNF410), ATF4, and Speckle-type POZ protein (SPOP) have all shown to play respective roles in the switch from HbF to HbA and merit further studies using CRISPR (20, 21).

BCL11A encodes a zinc-finger protein which mostly functions as a repressor and is involved in fetal-to-adult hemoglobin switching (16). By directly binding to a TGACCA motif at −115 bp of the γ*-globin* promoter it plays a crucial rule in silencing expression of the γ-globin genes (22). This promotes the formation of adult hemoglobin (HbA) over HbF (23). An interesting extension of this is KLF1, which also plays a role in fetal-to-adult hemoglobin switching by activating BCL11A as well as promoting HBB directly (24).

HBG1 and HBG2 encode for the γ-chain of HbF, they are homologous except for some differences in the upstream region of their respective distal promoters (25). In order to promote the transcription of these genes, CRISPR/Cas9 was used to knockout parts of the promoter region where repressors would bind. The inspiration for this method came from nature in the form of hereditary persistence of fetal hemoglobin (HPFH), a benign genetic condition in which HbF production persists into adulthood (26).

ZNF410 does not directly bind to HBG1/2, instead, it binds the Chromodomain-helicase-DNA-binding protein 4 (CHD4) gene. In turn activating the CHD4/NuRD (nucleosome remodeling and deacetylase) complex which is used in the repression of HBG1/221) What makes ZNF410 unique to some of the other HBG1/2 repressors is that it is highly specific for the CHD4 gene, to such a degree that it’s two binding site clusters are not found elsewhere in the genome (20, 27).

SPOP is a substrate adaptor of the CUL3 ubiquitin ligase complex (28). It acts independently of BCL11A in suppressing HBG1/2 and when depleted was shown to amplify the effects of hydroxyurea and pomalidomide, pharmacological inducers of HbF (29). It is suspected that SPOP-CUL3 promotes the ubiquitination and degradation of transcription factors that activate HBG1/2 (29).

As CRISPR research continues to advance, the number of publications available can be daunting to read through. A meta-analysis allows us to take the data from multiple publications and create a clearer image of the results. Even among studies which may seem conflicting a meta-analysis can yield statistically significant results, said results will also have a better ability to be extrapolated to the larger population.

This meta-analysis would allow us to obtain a larger view of the effects that CRISPR targeting these genes can have on HbF production. While also comparing the potential weaknesses of the different targets, such as the likelihood of producing off-target mutations. This will help to create a clearer path to guide future research in regard to which genes merit further research and which should be discounted.

## Materials and methods

### Identification of eligible studies

A comprehensive examination of all peer-reviewed studies published through June of 2021 was performed using PubMed. Combinations of the search terms “β-Thalassemia,” “beta-thal” “sickle cell disease,” “SCD,” and “CRISPR” were used to screen for studies for potential inclusion. The searches were completed with no language restrictions and no additional search engine filters.

### Inclusion and exclusion criteria

**FIGURE 2:**
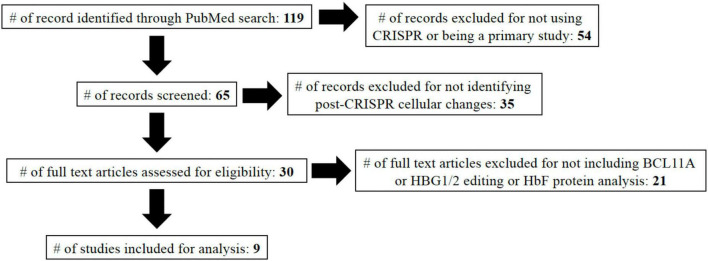
PRISMA diagram. In total, 119 studies were identified using the search terms: “β-Thalassemia,” “beta-thal,” “sickle cell disease,” “SCD,” and “CRISPR.” Studies were then included/excluded based on described characteristics. The nine studies included in the study are referenced (13, 17–19, 25, 30–33).

The following exclusion criteria were applied: non-primary studies like reviews, letters to the editor, conference abstracts, studies that did not contain specific CRISPR gene edits, studies without available full-texts, and studies that did not measure any post-CRISPR edit cellular changes. Final inclusion criteria included: assessment of HbF protein levels following CRISPR edit compared to control cells and studies that included CRISPR editing of either or both of BCL11A and HBG1/2. A detailed PRISMA diagram is included in Figure 2.

### Data extraction

Hemoglobin expression, n number, and corresponding SD values were collected from texts and/or figure legends of studies where included. Studies that did not explicitly indicate mean and/or SD values were extrapolated *via* pixel analysis of relevant graphs using ImageJ. Pixel sizes were determined using two known points (e.g., the distance between 0 and 10% on the *y*-axis is 200, then 1 pixel = 0.05%). Then the pixel distance between a zero value and an unknown value was determined and multiplied by the pixel size (e.g., the distance between 0% and the unknown mean was 50,000, 50,000/200 = 250; 250*0.05% = 12.5%). This method was performed on the following studies: Wu et al. (17), Khosravi et al. (18), Traxler et al. (30), Martyn et al. (31), Li et al. (32), Lamsfus-Calle et al. (25).

### Statistical analysis

The standardized mean difference (SMD) was used to quantify effect sizes of combined studies using a random effects model. For this study SMD score of 0.2–0.5 were considered small, 0.5−0.8 medium, and values > 0.8 were considered large. Nevertheless, we conducted and compared both common-and-random-effect models. We employed exact computations for the bias factor to evaluate the studies for heterogeneity, chi-square tests and I^2^ statistics were utilized. A value of 0.05 was considered statistically significant for the chi-square tests and I^2^ values ≥ 75% was indicative of high heterogeneity. Subgroup analysis was performed between BCL11A and HBG1/2 genes. R (version 4.1.2) was employed for all data analysis.

## Results

### Analysis of literature search

Following application of all exclusion criteria, 30 studies remained that analyzed post-CRISPR edit cellular changes. In an effort to focus on clinical significance of changes, we chose to focus only on studies that included HbF protein expression analysis over those that looked at only mRNA expression. Multiple genes including BCL11A, HBG1/2, and KLF1 had two or more studies analyzing HbF protein expression. However, we opted to only include BCL11A and HBG1/2 for analysis since the lack of studies including KLF1 posed a significant obstacle to sufficient comparative analysis (Figure 2).

Post-CRISPR editing HbF values from the included studies came from a variety of *in vitro*, *in vivo*, and *ex vivo* sources. Analysis of protein levels was determined *via* high-performance liquid chromatography in all studies except for Khosravi et al. (18) (western blot) and Mingoia et al. (33) (flow cytometry). HbF values were determined as a percentage of HbF of total hemoglobin production. In order to collect a sufficient amount of data, all studies regardless of cellular source were included. To control sample source variability, a random effects model was applied during the meta-analysis and forest plot generation.

### Meta-analysis results

**FIGURE 3:**
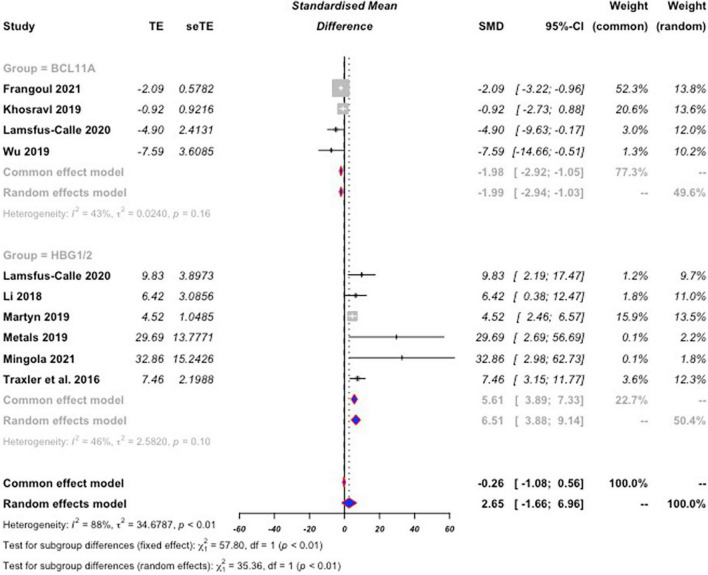
Forest plot for BCL11A and HBG1/2. Standard mean difference (SMD) values were calculated for both BCL11A, and HBG1/2 based on percent HbF expression as a total of hemoglobin expression following CRISPR editing compared to baseline. SMD values are negative for BCL11A not as an indication that expression levels dropped, but as a differentiator from the positive values of HBG1/2. The calculated SMD values from the collection of BCL11A studies was then compared to the calculated values from HBG1/2 for comparison of efficacy of HbF expression induction. The random effects model was used due to significant heterogeneity between the included studies. Editing of HBG1/2 significantly increased HbF expression by comparison to BCL11A editing (*p* < 0.01).

There was significant heterogeneity between the studies (I2 = 87.6% [95% CI: 79.1%,92.6%], *p* < 0.01); therefore, we used a random-effect model to estimate mean differences. Statistical results reveal that both the BCL11A and HBG1/2 showed significant increases in HbF protein expression post-CRISPR editing compared to controls with standardized mean differences of −1.98 [95% CI of −2.94, −1.02] and 6.51 [95% CI of 3.87,9.14], respectively (Figure 3). This indicates that a significant effect in HbF induction was achieved by comparison to baseline expression levels. A significant difference was also found between BCL11A and HBG1/2 (*p* < 0.01), suggesting that CRISPR editing of HBG1/2 may have more effect on the induction of HbF protein expression than BCL11A.

## Conclusion

In this study, we considered 119 studies for inclusion for the study. Following the screening based on the exclusion and inclusion criteria, the protein expression of HbF in 9 individual studies utilizing CRISPR editing of either or both of BCL11A and HBG1/2 were analyzed. It was determined that CRISPR editing of both BCL11A, and HBG1/2 significantly induces HbF expression by comparison to control values. It was also identified that HBG1/2 may have a more potent induction of this HbF protein expression by comparison to BCL11A (*p* < 0.01) (Figure 3). One limitation of this study was the sheer lack of available CRISPR data for SCD and β-Thalassemia. Due to CRISPR technologies being less than a decade old, much is yet to still be discovered and investigated with its use. More studies on these and other genes in the treatment/management of SCD and β-Thalassemia are required to further elucidate the true significance of CRISPR therapy.

While this analysis may have identified that HBG1/2 CRISPR editing is more effective than BCL11A editing, it is important to note that CRISPR editing of BCL11A is the only therapy that has progressed to human trials. Frangoul et al. (13) examined the use of BCL11A editing in two separate patients (one with SCD and one with β-Thalassemia). Both patients were transfusion dependent prior to receiving the therapy, however, following treatment, both patients had successful elevation of their long-term hemoglobin levels to become transfusion independent. Additionally, the SCD patient reported zero vaso-occlusive events over the 16-month period of the study after averaging 7 such events in previous years. The findings from this study highlight the potential efficacy of CRISPR gene editing in the treatment of SCD and β-Thalassemia, as well as underscore the importance of expanding on HBG1/2 gene editing in the future.

As mentioned above, there are multiple other genes that have been and/or are under investigation for potential efficacy of CRISPR editing in SCD and β-Thalassemia. More investigation of these genes such as KLF, ZNF410, ATF4, and SPOP and how CRISPR editing can affect HbF protein levels needs to be performed to elucidate additional targets that may be more efficient or effective than BCL11A or HBG1/2. Additionally, the gold standard for treatment and cure of SCD and β-Thalassemia is likely to be successful editing and correction of defective beta globin genes. While there are a few studies that have begun to investigate this possibility, it is still in its infancy and very few post-edit findings are currently available.

A major concern about the efficacy of CRISPR gene editing is the balance of effective gene editing with cellular toxicity. One possible way to mitigate these concerns is the utilization of nanoparticles for CRISPR delivery. Cruz et al. (34) was able to maintain a high level of CRISPR editing (elevation of gamma globin mRNA levels to 47–69% from 15 to 16%) while significantly reducing cellular toxicity. Increased efficiency of delivery *via* nanoparticle technologies could be critical to increasing overall efficiency of CRISPR gene editing in a clinical setting.

Additional benefits to increasing efficiency of CRISPR editing and reduced cellular toxicity could include the ability to therapeutically target multiple genes simultaneously. As mentioned above, there are multiple genes that play a role in HbF protein expression. Therefore, it is possible that by CRISPR editing multiple genes simultaneously, there could be a synergistic induction of HbF expression that could have an added clinical benefit. Thus, targeting multiple genes concurrently requires investigation at the pre-clinical level for efficacy.

In addition to studying CRISPR-complex delivery mechanisms for gene editing efficacy, it is also important to consider varying patient genetics and how they can have an effect as well. Modari et al. (35) identified that even within the same patient samples, variations in homology directed repairs can cause wide variations in responses to CRISPR directed gene editing. Therefore, significant study into how patient genetics can play a role in their response to CRISPR editing is required in order to improve overall efficacy of therapies.

Though in its infancy, CRISPR editing in the treatment of diseases has already shown excellent progress and effectiveness. In this meta-analysis, it was identified that CRISPR editing of HBG1/2 offers a possible benefit over BCL11A for induction of HbF expression for the management of SCD and β-Thalassemia.

Advancements in CRISPR gene editing technologies highlight a promising movement into the future of medicine. However, they do not come without significant ethical challenges and concerns. Brokowski and Adli (36) sought to discuss some of these ethical issues by focusing on four key aspects: (1) the extent to which CRISPR use should be permitted; (2) access to CRISPR applications; (3) what regulatory frameworks should be put into place for clinical research; and (4) what international regulations can be employed to govern the inappropriate utilization of CRISPR. They concluded that moral decisions and governing bodies regarding CRISPR utilization should be constantly evolving with the changing scientific landscape. Currently, governing bodies on the use of CRISPR technologies are still limited, and those that do exist like the International Commission on the Clinical Use of Human Germline Genome Editing are still hesitant on the application of this technology for therapeutic use. Continued discussion on the ethical issues and the balance of therapeutic applications of CRISPR should remain continuous as the technology becomes more expansive.

## Data availability statement

The original contributions presented in this study are included in the article/supplementary material, further inquiries can be directed to the corresponding authors.

## Author contributions

AQ analyzed the initial manuscripts, performed the analysis, and wrote the manuscript. DA wrote the manuscript. PH performed the analysis and wrote the manuscript. SP conceptualized the study, performed the analysis, and wrote the manuscript. All authors contributed to the article and approved the submitted version.
